# Reconceptualizing Social and Emotional Competence Assessment in School Settings

**DOI:** 10.3390/jintelligence11120217

**Published:** 2023-11-22

**Authors:** Nathaniel von der Embse, Stephen Kilgus, Carly Oddleifson, Jason D. Way, Megan Welliver

**Affiliations:** 1 Department of Educational and Psychological Studies, University of South Florida, Tampa, FL 33620, USA; meganwelliver@usf.edu; 2 Department of Educational Psychology, University of Wisconsin, Madison, WI 53706, USA; skilgus@wisc.edu (S.K.); oddleifson@wisc.edu (C.O.); 3 Renaissance Learning, Wisconsin Rapids, WI 54494, USA; jason.way@renaissance.com

**Keywords:** school mental health, assessment, social emotional learning

## Abstract

The mental health needs of youth are both significant and increasing. Recent advancements have highlighted the need to reduce psychological distress while promoting the development of important social and emotional competencies. Current social and emotional assessment tools are limited in important ways that preclude their widespread use. In the current article, these limitations are discussed. A novel social and emotional learning assessment framework guided by methodological and theoretical innovations is presented. Future research directions and opportunities are discussed.

## 1. Reconceptualizing Social and Emotional Competence Assessment in School Settings

The need to support student mental health, well-being, and social and emotional development is clear and urgent. The recent pandemic has highlighted students’ widespread and acute mental health needs in the United States (Office of the Surgeon General’s Advisory 2021). While neither the negative impact of significant environmental events on mental health (e.g., Hurricane Katrina; Osofsky et al. 2015) nor the necessity of student mental health services (Twenge et al. 2019) are new phenomena, emerging research has demonstrated the extensive influence of the pandemic on adolescent well-being over multiple years (De France et al. 2022). Social isolation, trauma over loss, and uncertainties regarding health risks from the pandemic have been linked to lowered youth mental health (Galea et al. 2020). The significant youth mental health needs made clear during and after the pandemic have brought new attention to the critical social and emotional competencies needed to succeed in a variety of environments and the critical role of schools in facilitating access to mental health, and social and emotional services (Rosanbalm 2021).

Over the last twenty years, schools have increasingly become the de facto provider of mental health services for youth, as community agencies have struggled to meet demand (Ali et al. 2019). Consequently, increased resource utilization has strained school mental health supports (Weir 2020). Despite recent government efforts advancing new funding for school mental health supports (United States Department of Education 2022), a critical shortage of school mental health professionals is unlikely to be remediated in the coming years (Whitaker et al. 2019). These personnel shortages have necessitated a reconceptualization of how mental health services are provided in school settings (Herman et al. 2020). Public health models offer promise in reducing the burden of significant mental health concerns. By utilizing prevention-oriented and tiered service frameworks with a focus on positive and prosocial skill development (e.g., Interconnected Systems Framework; Barrett et al. 2013), the development of later substantial mental health concerns can be diminished (Taylor et al. 2017). 

Schools have increasingly adopted universal programs and curricula to support student social and emotional development within a tiered framework (Eklund et al. 2018). Decades of research have supported the effectiveness of these efforts as linked to improved academic performance in addition to long-term social, behavioral, and academic benefits (Durlak et al. 2011). Social–emotional learning (SEL) is one such framework that has been widely adopted by various state education agencies with defined standards for learning (Collaborative for Academic, Social, and Emotional Learning CASEL 2019). Defined broadly, SEL refers to “the processes through which children and adults acquire and effectively apply the social and emotional knowledge, attitudes, and skills necessary to understand and manage emotions, set and achieve positive goals, feel and show empathy for others, establish and maintain positive relationships, and make responsible decisions.” (CASEL 2019). Given the ubiquity of SEL in schools, social and emotional programming has increasingly become synonymous with SEL. However, installing social and emotional support within a tiered system is not without challenges. For example, significant differences in parental support for SEL are split along partisan political lines, despite a widespread agreement on the basic tenets of SEL when explained with examples to parents (i.e., “support for the idea, but not the label”, Tyner 2021). 

The confusion and miscommunication of social and emotional skills, and competencies more broadly, are not limited to parents. The National Research Council (2011) noted substantial overlaps in various definitions of social–emotional skills with various personality dimensions across curricula, and the potential of a “jangle fallacy” in using different terminology to describe the same construct (see Kelley 1927). Relatedly, there is a potential for using the same terms to describe different constructs (jingle fallacy) that have been noted in the assessment of other psychological constructs (e.g., test anxiety; Putwain et al. 2020). The differing definitions within the social and emotional domains may be particularly problematic when developing assessment tools with evidence of construct validity. Without clear and agreed-upon definitions, two “social-emotional assessment tools” could be measuring two very different constructs (Cronbach and Meehl 1955). Precision and consistency are essential to advance assessment and, ultimately, the understanding of social and emotional competencies. As such, we use “social and emotional competency” as a non-framework or non-curriculum-specific term to encompass the social and emotional knowledge, skills, and attitudes that are necessary for long-term success. In the following article, we delineate some of the pressing challenges in social and emotional assessment and offer a vision for new opportunities in social and emotional assessment. 

## 2. Changes in Social and Emotional Assessment

There has been a significant and relatively recent paradigm shift in the field of social and emotional assessment from a focus on clinically derived tools that solely measure psychological deficits (e.g., Child Behavior Checklist, CBCL; Achenbach 1991) to those that incorporate student strengths (e.g., Strengths and Difficulties Questionnaire, SDQ; Goodman 1997). This shift has been largely informed by innovations from the field of positive psychology (Seligman 2011) and is represented in the dual-factor model of mental health (Greenspoon and Saklofske 2001; Suldo and Shaffer 2008). This model defines complete mental health as consisting of a limited display of psychological problems as well as the presence and development of prosocial skills. This modern conceptualization of mental health has had wide-reaching implications for assessment development, with new tools including a focus on malleable social and emotional competencies that are informative of interventions rather than a singular focus on psychological problems. New social and emotional assessment tools should assess *needs* (rather than just problems) and include the perspectives of multiple informants (von der Embse and De Los Reyes 2023).

A second, and related, paradigm shift reflected an evolution of how psychological domains were conceptualized from categorical to dimensional (De Fruyt and De Clercq 2014). For example, rather than seeking to diagnose and categorize, newer assessments often reflect the *degree* of need within particular domains. This may be important for school decision-makers to prioritize services with a focus on malleable characteristics rather than static deficits. Advancements in statistical modeling techniques (e.g., diagnostic classification modeling; Rupp and Templin 2008) allow for the evaluation of within-item dimensionality, whereas an item may be reflective of several latent factors, ultimately permitting the development of assessment tools that are both brief and measuring of multiple domains (see Kilgus et al. 2020). In sum, these paradigm shifts have further highlighted the need to critically evaluate how current and future social and emotional assessment tools reflect (1) the *needs* of youth from a dual-factor model and (2) identify *malleable* characteristics to inform service delivery. To do so effectively, and as noted earlier, the need for consistent definitions of social–emotional constructs has led to the development of social and emotional competence taxonomies. 

## 3. Defining Social and Emotional Competencies

Substantial empirical literatures on “non-academic” skills include terminology such as character traits, soft skills, and social–emotional skills (Duckworth and Yeager 2015). For example, CASEL defines social and emotional competency as an umbrella term that encompasses skills, attitudes, and knowledge and is represented in five broad social and emotional domains: self-awareness, social awareness, self-management, relationship skills, and responsible decision-making (CASEL 2019). These competencies include domains (e.g., identifying one’s emotions) and also subdomains of more specific skills designed to meet a standard and/or benchmark. Additional models identify similar (e.g., self-management) yet somewhat distinct domains (e.g., open-mindedness and negative-emotion regulation; Primi et al. 2016), while others are informed by positive psychology (5Cs; e.g., confidence and compassion; Bowers et al. 2010; PERMA, Seligman 2011). Even the number of domains is subject to variability, as the National Research Council (2011) identified only three domains, which are cognitive, interpersonal, and intrapersonal skills. Given these multiple and various definitions, the Taxonomy Project (Jones et al. 2019) sought to develop a comparative method for identifying similarities and differences across various social–emotional frameworks and included six domains and 23 subdomains. The resulting taxonomy allowed for an evaluation of definitions used to describe various social and emotional competencies across frameworks, thus reducing the potential for a jingle-jangle fallacy. These efforts have now been reflected in various state standards and policies (Dermody and Dusenbury 2022). 

## 4. Attempts to Categorize Developmental Expectations for Social–Emotional Competencies

**Figure 1:**
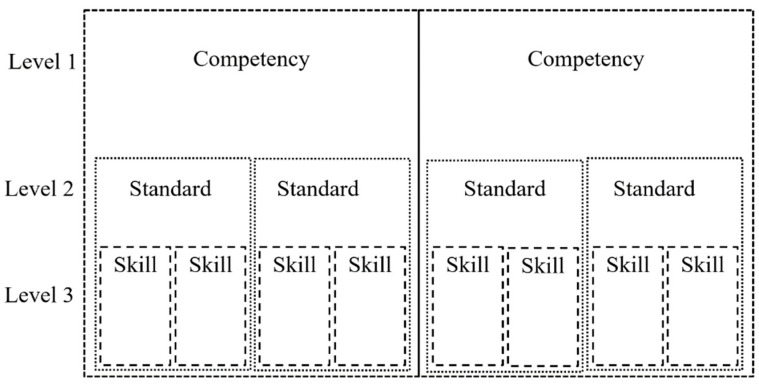
Social and emotional policy hierarchy of competencies, standards, and skills.

Most states have policies that specify standards to teach social emotional competencies that align with the CASEL framework (Eklund et al. 2018). The CASEL framework consists of five core competencies that are important at all ages. Competencies are the first level of many policy frameworks (Level 1 of Figure 1) and inform our later conceptualization of a new social and emotional competency assessment. Implicit in this CASEL framework and the corresponding state policies is the concept of developmental change and developmental periods. Developmental periods are qualitatively distinct life stages (e.g., early childhood and adolescence). These developmental periods include stage-specific developmental tasks. The importance of considering age-differentiated developmental tasks was highlighted in a Special Issues brief published by the Assessment Work Group (AWG)—a working group affiliated with CASEL and the American Institute of Research (AIR) (Denham 2018). An example of an elementary-age children’s developmental task is to demonstrate emotional regulation with the peer group, showing emotions in appropriate contexts (Denham 2018). These developmental tasks are what a particular culture expects from individuals (Denham et al. 2009); thus, developmental tasks vary across cultures. Unfortunately, many different terms are used to refer to these developmental tasks. School psychologists sometimes refer to these developmental tasks as developmental milestones (Whitcomb and Feuerborn 2020). Policy-makers refer to these indicators as standards, indicators, or benchmarks, whereas SEL experts call them developmental benchmarks (e.g., the proficiency levels of specific knowledge and skills; Dusenbury et al. 2014). 

**Figure 2:**
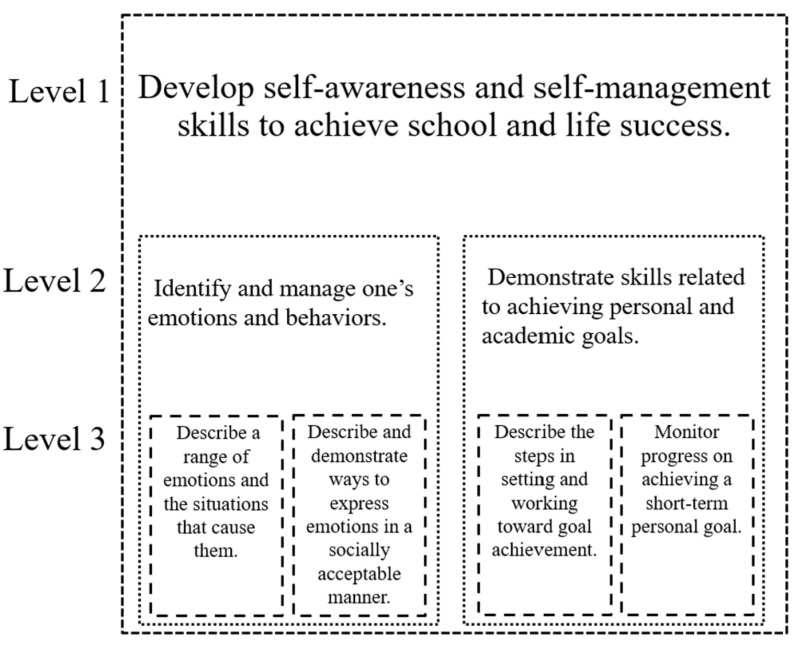
Example of Illinois’ social and emotional hierarchy.

In this article, we refer to developmental tasks as standards (Level 2 of Figure 1). Experts recommend these standards at every grade level. In other words, the same CASEL 5 competencies are present at every grade level; while each grade level has its own standards, students use different skills to meet these standards. For this reason, some policy frameworks include developmentally appropriate skills nested within the standards (Level 3 of Figure 1). Figure 2 contains language pulled from a small section of the SEL policy in the state of Illinois (Illinois State Board of Education n.d.). At the first level of the Illinois framework, the competencies of self-awareness and self-management are grouped together. At the second level, there are two standards: (1) identify and manage one’s emotions and behaviors; (2) demonstrate skills related to achieving personal and academic goals. At the third level are four different skills. Note that while many frameworks do contain competencies, standards, and skills, there is still great variability across different state policy frameworks (Dermody and Dusenbury 2022). 

A recent systematic review of SEL policy indicated that there is a lack of SEL assessments that could be utilized to measure SEL development across K-12 (Eklund et al. 2018). There are limited SEL assessment tools exhibiting the psychometric evidence necessary to support data-derived treatment or classification that may, in turn, restrict the empirical evidence necessary to inform policy-makers on developmentally appropriate SEL expectations (McKown 2019). While many of the skills presented by states are not empirically based (Eklund et al. 2018), some researchers are trying to produce empirical evidence that would inform policy on social–emotional benchmarks. More recent systemic efforts have utilized the EASEL Lab’s Taxonomy Project comparison tools to propose benchmarks for social and emotional competencies across grades (Soland et al. 2022). However, these benchmarks have not been replicated across multiple years and in new samples such that the stability of constructs (i.e., benchmarks) in any one year has yet to be confirmed. Taken together, the challenges of consistently defining social and emotional competencies, and the varying expectations of development across ages, underscore the importance of new assessment methods that measure along a developmental continuum. 

## 5. Theoretical Innovation: Dynamic Skill Theory

As depicted in Figure 1 and Figure 2, competencies consist of different skills. At present, there is no single unifying theory that explains social–emotional development. The process by which social–emotional competencies and skills develop and the consistent developmental expectations have yet to be agreed upon. However, similar to how reading intervention and assessment are grounded in a basic science about how children learn to read, social–emotional assessment should be grounded in a basic science about how students develop social and emotional skills. While the CASEL-5 framework explicitly highlights *what* social–emotional competencies are important, this framework does not depict *how* students develop the skills that constitute social–emotional competence. Social and emotional researchers have made great progress advancing theories on the development of specific social–emotional competencies such as social skills (e.g., the Social Development Model; Hawkins et al. 2004), self-regulation (e.g., Zimmerman and Schunk’s (2011) model of self-regulation development), and emotional intelligence (e.g., multi-level investment model; Zeidner et al. 2003). However, these theories are frequently too narrowly defined in their focus. On the other hand, while many broader theories are associated with social and emotional approaches (e.g., social learning theory, behavior analytic theory, and ecological systems theory), no single theory has emerged as the most widely accepted or understood (Whitcomb and Feuerborn 2020). Ecological systems theory has been criticized for not adequately addressing development change (Lourenço 2016). In contrast to ecological systems theory, dynamic skill theory (Fischer 1980; Bidell and Fischer 2006) addresses context and developmental change. In contrast to the three aforementioned narrowly defined theories, dynamic skill theory is broad and can be applied to understand the development of many types of social–emotional competencies.

Pioneering developmental psychologist Jean Piaget laid the foundation for dynamic skill theory. When researching cognitive skill acquisition, Piaget introduced the cognitive stage model and explained how individuals develop more complex skills over time. Dynamic skill theory is an expansion of Piaget’s stage model because Fischer highlighted specific age ranges (i.e., stages) where individuals typically reach certain skill levels (Mascolo and Fischer 2015). However, while Piaget did not think that context plays a large role in development, Fischer asserted that context is hugely influential in development and that skills are ultimately a property of both person and context (Fischer 1980; Bidell and Fischer 2006). The context that a person is residing in greatly influences their level of functioning and skill use. Fischer’s dynamic skills theory aligns with the concept of far transfer in learning theory. Far transfer occurs when a student applies learning to a situation that is quite different from the situation in which they learned the skill (Barnett and Ceci 2002) Dynamic skill theory states that in addition to being context-dependent, skill development is non-linear. This means that individuals can progress and regress in their use of adaptive skills. Given this non-linearity, Fischer depicted skill development as a developmental web in which children move up and down on strands on a web depending on whether they are progressing or regressing (Fischer 1980). Developmental webs vary across children and represent a child’s own unique developmental pathway. The shape of a developmental web is affected by context (Fischer 1980).

Some social–emotional experts have explained the development of self-regulation skills in a manner consistent with dynamic skill theory. Although they do not explicitly espouse dynamic skill theory, their conceptualization of the development of self-regulation does offer evidence for the potential value of using dynamic skill theory as a framework. More specifically, researchers at The Ecological Approaches to Social Emotional Learning (EASEL) Laboratory at Harvard shared that “children do not master this body of skills linearly but instead go through gradual cycles of progression and regression, needing to learn and re-learn skills under new and different circumstances” (Bailey et al. 2019, p. 9). The complexity of a child’s social–emotional competencies must change over time to adapt to increasing environmental demands, and the level of support within an environment may influence the ability to display said skills (Bailey et al. 2019). Beyond the theoretical work completed at the EASEL Lab, empirical research on social–emotional competency development suggests that dynamic skill theory may be an appropriate theoretical framework for social–emotional assessments. More specifically, Ayoub et al. (2011) utilized dynamic skill theory to depict the developmental pathway to integrated social skills. They used multi-level models and three-level growth models to analyze a dataset that included 3001 Head Start families. The multiple mechanisms and mediating processes influencing the development of self-regulatory and language skills in children was explained by the researchers through the use of dynamic skill theory. Most relevant to social–emotional development were the results that revealed that Early Head Start protects self-regulatory development from the effects of parenting stress and demographic risks (Ayoub et al. 2011).

In summary, dynamic skill theory is an ideal theoretical underpinning for social–emotional assessments as it aligns with the way that social and emotional policymakers and experts conceptualize social–emotional development. Namely, youth progress through specific stages during which certain levels of development are reached. Combined with the CASEL conceptualization of five social and emotional domains, multi-level models offer an important advancement in understanding the hierarchical, context-dependent, and non-linear development of social emotional skills within a broader social and emotional competency. Social–emotional skill assessments could have the strong theoretical underpinning of dynamic skill theory; whereas, during our review of social–emotional theories, we could not find a theory that could underpin social–emotional assessments. In other words, there is theoretical support for assessing social–emotional skills and we could not find theoretical supports for assessing SEL at the level of competencies.

## 6. Critique of Existing Social–Emotional Competency Assessment

The limitations of broader assessment methodologies and specific assessment tools further underscore the need for novel approaches to social and emotional assessment research. The two broader categories of social and emotional assessment methodologies include rating scales and performance tasks (Duckworth and Yeager 2015). *Rating scales* require informants (e.g., teachers, parents, or students) to provide their perceptions regarding a range of student skills, behaviors, knowledge, and attitudes. Typically, this is completed by the informants using some quantitative and ordinal scale (e.g., a 5-point Likert scale) to indicate either (a) the extent to which a series of statements (i.e., as described in items) are true of a target individual or (b) the frequency with which the target individual displays the behavior, emotion, or quality described in the statements. *Performance tasks* involve students encountering contrived situations designed to elicit one or more social and emotional skills. Some performance tasks require in vivo responses, such as those founded upon role-play scenarios. Other performance tasks are technologically mediated, requiring individuals to respond within a virtual scenario or interpersonal interaction. Performance task situations are intended to elicit meaningful variation in skill performance across students, permitting differentiation of students relative to their level of the skill in question. Some performance tasks are conspicuous in nature, transparently providing details and contextual cues related to the social and emotional skills they are assessing. Other tasks are more inconspicuous or “stealth”, as they collect information about specific skills without such transparency (e.g., observing and recording a student response within a naturalistic social situation). 

Multiple research teams have compiled and reviewed social and emotional skill assessments. Recently, the Collaborative for Academic, Social, and Emotional Learning (CASEL 2019) developed the *SEL Assessment Guide*, which includes a review of each measure’s foundational constructs, administration and scoring procedures, and psychometric evidence. A review of the assessment tools within this guide and the literature supporting these tools reveal notable challenges. Each of these challenges is described in further detail below. 

### 6.1. Evidence to Support Use Limitations

The first limitation pertains to the nature of the evidence supporting each measure, as summarized in the SEL Assessment Guide. Though many reviewed measures are supported by strong reliability and validity evidence, such evidence has its limitations. Though this evidence can speak to how assessment scores should be *interpreted* (e.g., with regard to the constructs for which it provides information), it does not indicate how the assessment should be *used* (Kane 2013). Evidence for assessment use is needed to guide practitioners on the defensible ways a tool can be applied within schools. Many schools are adopting multi-tiered systems of support (MTSS) through which services are provided across a continuum of intensity to meet varying student needs, including those within the social and emotional domains. Within MTSS frameworks, assessments are commonly used for one of the following purposes: (a) *universal screening*, or the systematic evaluation of a broader population (e.g., school) to identify those possessing some characteristic (e.g., lagging social and emotional competencies); (b) *program evaluation*, or the collection of data to examine the influence of some program (e.g., school-wide SEL instruction); (c) *progress monitoring*, or repeated assessment over time to evaluate an individual’s response to an intervention; and (d) *problem analysis*, or the use of data to inform intervention planning, such as through matching students to an intervention or adapting intervention components to align with student needs or characteristics (von der Embse et al. 2022). 

A review of the literature reveals several social and emotional assessments possessing evidence supporting their use in universal screening or program evaluation. Measures supported for universal screening typically include brief rating scales of approximately 25 items or less. Examples include the *Devereux Student Strengths Assessment—mini* (DESSA-mini; Naglieri et al. 2011) and the *Social Skills Improvement System—Social and Emotional Learning Brief Scales* (SSIS SEL*b*; Anthony et al. 2020). Other measures have been supported for use in program evaluation, particularly (a) comprehensive rating scales, such as the full DESSA (LeBuffe et al. 2009) and SSIS SEL measures (Gresham and Elliott 2017), as well as (b) performance tasks, such as the SELweb system (McKown et al. 2023). 

In contrast, despite developer claims, research has yielded far fewer social and emotional assessment tools with support for use in progress monitoring and problem analysis. To be suitable for use in progress monitoring, tools should possess evidence of change sensitivity—that is, the capacity to document growth in social and emotional skills over time (Gresham et al. 2010). Progress monitoring can be specifically challenging within social and emotional assessments given the wide variations in typical developmental expectations. Measures should also be hierarchically organized such that they can be used to track student growth across increasingly complex competencies aligned with developmental expectations (Abrahams et al. 2019). To be suitable for use in problem analysis, a tool should possess evidence of its ability to both (a) differentiate between the social and emotional competencies a student has mastered and those they are still building (Kilgus et al. 2015), and (b) inform intervention, such that intervention outcomes are more positive when informed by the assessment tool (i.e., treatment utility; Nelson-Gray 2003). The lack of research for both progress monitoring and problem analysis establishes the need for such evidence in relation to existing tools, as well as the development of new measures specifically designed for such uses.

### 6.2. Technology-Based Social and Emotional Assessment Limitations

There have recent novel advancements in the use of technology for social and emotional assessment (Russo-Ponsaran et al. 2022). To date, the majority of social–emotional assessments have required an active informant response to test content that is consistent across administrations (e.g., the same 40 items are completed for each student) and conspicuous in their evaluation of social–emotional skills. An increasing number of assessment methods permit (a) passive data collection, which reduces informant effort, or (b) inconspicuous content, which can reduce the likelihood of informants “faking good” or providing an overly positive depiction of the assessment target. The majority of these methods represent technology-based assessments that are web or app-based, may contain animations or videos, or may be more sophisticated and include advanced technology like digital biosensors. The advantage of technology-collected data is that they can reduce the burden of data collection that falls on already busy school staff. Stealth assessments have the potential to increase the validity of social and emotional assessments because they remove the possibility of test-taking engagement threatening the validity of the interpretation of the results (Stoeffler et al. 2022). Stealth assessment is non-obstructive and can be a type of game-based assessment. Though such assessments are occasionally characterized by relatively limited ecological validity, they can nevertheless yield information predictive of social–emotional functioning. More research is needed on stealth assessments so that they authentically represent real-world environments. Another example of a technology-based social and emotional assessment is an online story-based assessment instrument to measure social and emotional growth in children (Fatolapour et al. 2014). Computerized adaptive testing (CAT) systems have been used to test for constructs such as depression (Grunebaum et al. 2021; Iwata et al. 2016) and schizotypal traits (Fonseca-Pedrero et al. 2013), but have not yet been used to measure social and emotional competencies. A primary advantage of using CAT assessments is increased efficiency and usability, with reduced completion time compared to traditional long-form and/or paper-based assessments (Gershon 2005). 

### 6.3. Methodological Limitations

An additional challenge with existing social and emotional assessments relates to the limitations of the methodologies. Research has repeatedly indicated that rating scale scores can be characterized by bias related to student gender and race/ethnicity (Ormiston and Renshaw 2023; Splett et al. 2020). It can be challenging for an informant (e.g., teacher, caregiver, or student) to accurately recall student behavior over an extended time frame (e.g., weeks or months), likely resulting in informants imposing their bias in recording their perceptions of the behavior. The potential for bias is further enhanced in the context of rater fatigue, which can diminish informant attention to the rating process (Myford and Wolfe 2003). Comprehensive rating scales require sustained time to complete, commonly taking 15–30 min per administration. Brief rating scales take less time but can prove cumbersome when completed repeatedly as part of progress monitoring or universal screening, particularly when raters have to complete the same set of items over time (Brann et al. 2021). Finally, the utility of a rating scale is inherently tied to its design. Each rating scale affords information regarding a set of social–emotional competencies, which it has operationalized in a unique way. A rating scale’s utility for each user is driven by alignment between the afforded constructs and the user’s unique service delivery needs.

Challenges are also inherent to the performance task method. Performance tasks can provide more objective information, potentially less characterized by perception-based rating scale data biases. However, performance tasks with sufficient empirical support within the literature are frequently both costly and time-intensive (e.g., McKown et al. 2023). Furthermore, the design of performance tasks can be complex, requiring the design of tasks that are (a) relevant to a diverse body of youth and (b) specific to students across multiple developmental levels, and aligned with their lived experiences, thus eliciting the intended social and emotional responses despite the contrived circumstances. Their repeated use at scale (e.g., across an entire school) for either universal screening or program evaluation could be cost and time prohibitive over time (Brann et al. 2021). When taken together, it is clear that innovation in assessment methods is needed to ensure high-quality data collection, interpretation, and use.

## 7. Methodological Innovations: Computer-Adaptive Testing

Classical Test Theory (CTT; Lord and Novick 1968) was the main method of analysis for social and emotional skill assessments before the advent of cost-efficient and more available computing power made more complicated analysis methods more feasible. CTT is primarily concerned with estimating a person’s unobserved true score on a test (T) based on an observed test score (X) and some random error of measurement (e):

X = T + e

While CTT does not require large sample sizes (*n* of around 100) to conduct analyses, item parameters can vary from sample to sample, and the probability of getting an item correct does not account for individual differences in ability related to the construct in question. In other words, it does not account for the fact that people with higher abilities are more likely to get an item correct than those of lower abilities relative to the sample. Furthermore, error in measurement can only be estimated as an average across different trait levels instead of errors at different, specific levels of the trait. Item response theory (IRT; Lord 1980) was developed to address some of these limitations. 

There are many different IRT models that can be used to estimate item difficulty, discrimination, and other parameters, but they generally are all concerned with estimating the probability (P) of responding correctly to an item (u_i_ = 1) given a particular examinee’s underlying ability (|θ = t):

P(u_i_ = 1|θ = t)

where theta (θ) represents an examinee-specific parameter indicating where a person falls on a latent (i.e., unobserved) trait continuum. Thus, the probability that IRT models provide is dependent on where a person falls on that continuum. People who are lower on the trait being measured will have a lower probability of getting an item correct, whereas people who are higher on that trait will have a higher probability of getting that same item correct. This is an advantage over CTT analyses, where the probability of getting an item correct is only based on the probability of getting it correct, regardless of the examinee’s ability. Another advantage is that the IRT item parameters should theoretically be the same regardless of the sample drawn from a population of interest, as opposed to CTT parameters. 

IRT methods allow for several applications that go beyond what is possible with CTT methods. One is the development of computer-adaptive testing (CAT) based on scores from IRT models, which holds a great deal of promise for assessing the *degree* of social and emotional skill mastery. Identifying the degree of mastery (as opposed to a mastered or not mastered dichotomy) is particularly important given the potential for non-linear growth in skills and the context dependency and subsequent variability of skill demonstration. CAT adapts the difficulty of the item, as aligned to the theoretical framework and related to the latent skill continuum, presented to the examinee to their estimated trait level ability that is estimated based on large-scale IRT analysis prior to the development of the CAT algorithm. This is only possible with IRT methods, as they take into account individual variations in trait level. For example, most traditional CATs of academic or cognitive skills start with a middle-difficulty item. If the examinee gets the item wrong, the next item presented is easier based on its item difficulty parameter. If the examinee gets the middle difficulty item correct, the next item presented is more difficult. Thus, the difficulty of the items presented to the examinee are adjusted until the proper trait level of the examinee is narrowed down to sufficient confidence and testing ends. 

There are several benefits of IRT-based CATs (Sereci 2003). One is that they utilize coverage of the entire latent skill continuum to ensure accurate measurement across all levels of the latent skill. Another is that CATs are more efficient to administer than non-adaptive tests. More accurate scores can typically be determined with fewer items administered, down to half of the items of a traditional non-adaptive test. This saves valuable time in school and classroom contexts. Similarly, CATs allow for greater test security because not all of the items are administered in any given session. Examinees will see different items from each other and aligned to an estimated skill level, so that the entire item bank is not exposed upon one test administration. However, this does mean that the overall item pool needs to be larger and refreshed occasionally, necessitating a larger investment in item development.

## 8. Novel Approach to Social and Emotional Competency Assessment

As noted above, several CATs have been developed to assess student academic achievement. Efforts have also begun in the mental health domain, with research demonstrating the potential promise of applying computer-adaptive testing principles to the development of computer-adaptive rating scales. There is the potential that computer-adaptive rating scales could address some of the limitations inherent in other social and emotional assessment methodologies, particularly those related to efficiency. A computer-adaptive process could reduce the time needed to collect social and emotional data. This increased feasibility might reduce rater fatigue, potentially limiting the rater bias introduced into rating scale data. This feasibility could also increase the overall efficiency of the social and emotional assessment process, increasing the likelihood that schools collect data to inform and evaluate their MTSS efforts. Moreover, there is an opportunity to reimagine social and emotional assessment as aligned to a theory-driven approach—such as the Dynamic Skills Theory aligned to a CASEL framework—that specifically recognizes the non-linear and context dependency of social and emotional development.

Our team has envisioned a novel multiple-gating approach to CAT-based social and emotional assessment that can be used to address multiple use types. The broader assessment system is designed to assess CASEL 5, with each factor corresponding to a different social and emotional competency: Self-Awareness, Social Awareness, Self-Management, Relationship Skills, and Responsible Decision-Making. Furthermore, given their relevance to school success, additional factors would relate to academic enabling skills that prepare students to access and benefit from academic instruction, including motivation, study skills, and academic engagement (DiPerna 2006). Research suggests that social and emotional variables are related but functionally distinct (Kilgus et al. 2020), with each contributing to the prediction of academic achievement (Kilgus et al. 2016). 

The first gate within the multiple-gating approach would represent universal screening. This gate utilizes a small subset of items (e.g., 8–15) that would be completed for all students in a population (e.g., school or district) to identify those needing additional support at an advanced tier to build their social and emotional skills. These items could represent the best predictors of overall social–emotional competence, as indicated by high factor loadings. The assessment system would include certain features to ward against rater fatigue resulting from completing the same universal screening tool multiple times per year over multiple years. Such a feature may include the random selection of these high-loading items from a broader pool of items, yielding an approach aligned with using multiple equivalent forms, as is available through the DESSA-mini. Student scores on these items would be aggregated to form an overall total score indicative of student social–emotional competence and aligned with the theoretical and conceptual framework. The resulting total scores would then be compared with empirically derived cut scores to determine which students likely need additional social and emotional support and should thus pass to the second gate. 

The second gate within the assessment system would then permit both (a) targeted screening to confirm student needs for additional support and (b) problem analysis to determine the nature of each student’s needs, informing intervention selection and adaptation. This second gate would represent a CAT, wherein the rating of each item (or groups of items depending on CAT design) would inform the selection and administration of subsequent items. The computer-adaptive approach would allow for the administration of a smaller number of items than a typical comprehensive rating scale, yielding a more efficient approach. The CAT might be made even more efficient if first-gate results could inform the CAT, suggesting which items should be prioritized for administration (e.g., given that they correspond to lower-performing subscales). To align with dynamic skill theory, the CAT should include a series of items within skill categories that are of varying difficulty and situated within the five CASEL social and emotional domains—that is, some items should represent simplistic versions of a skill (e.g., “I know right from wrong”), whereas others will represent more complex versions (e.g., “I consider the moral and ethical implications of my actions”). This hierarchical organization of items is intended to reflect the increasing complexity of how the same skill can be displayed across development. 

The CAT would yield a series of IRT scores corresponding to the aforementioned social and emotional competencies and academic enabler factors. Each score could be compared to empirically derived cut scores to determine which skills are below expectations and should thus be targeted for intervention. Multiple versions of the CAT might be developed, including those meant to inform Tier 2 intervention and those that inform Tier 3 intervention. The Tier 2 approach should likely be more efficient, given that it could be used with more students. Such efficiency might be found by fixing the terminating criterion to a less stringent value, while the Tier 3 approach would correspond to a more stringent value. 

We believe this proposed assessment system would evidence high levels of usability, characterized by both its acceptability and feasibility for educator use (Brann et al. 2022). A universal screening process used at Gate 1 would likely evidence the brevity needed for adoption and use at scale across a large number of students (Glover and Albers 2007). A CAT-based approach to problem analysis at Gate 2 would yield an assessment process that is briefer and more varied than the process of completing standard comprehensive rating scales, which involves the completion of the same set of numerous items across administrations. We hope this increased usability would translate to an increase in the adoption of social–emotional assessment in schools and its use to inform prevention and intervention efforts. 

## 9. Future Directions in Social and Emotional Assessment

Given the increased mental health needs present in schools, assessment practices must adapt to these changing needs. This includes the identification of social and emotional needs in a usable, efficient, and relevant manner. Moreover, social and emotional assessment needs a conceptual (CASEL) and theoretical (Dynamic Skills Theory) framework that informs a hierarchy of skills within a developmental process that is non-linear and context bound. As noted in the present article, a number of limitations to the most frequently used social and emotional assessment tools may limit their widespread adoption and use. Without such tools, schools may focus efforts on those students whose behavior may be disrupting instructional environments with less priority towards students who may not be exhibiting the social and emotional competencies needed for school success. There are numerous opportunities for innovation in developing social and emotional assessments to meet this critical need (McKown 2019). We present one such vision of a new social and emotional assessment framework to be used across the tiers of decision-making. To advance said vision, research will be necessary to validate how data are used, specifically focusing on the utility in making treatment decisions. The current state of social and emotional assessment suggests significant demand and a subsequent opportunity to offer novel solutions, ultimately leading to improved student outcomes. 

## Data Availability

No new data were collected or analyzed in study and thus data sharing is not applicable.
